# Family functioning and quality of life among children with anxiety disorder and healthy controls. A cross-sectional study

**DOI:** 10.1590/1516-3180.2018.0145240418

**Published:** 2018-08-13

**Authors:** Yusuf Öztürk, Gonca Özyurt, Aynur Akay

**Affiliations:** I MD. Assistant Professor, Department of Children and Adolescents, Bolu Abant İzzet Baysal Üniversitesi, Bolu, Turkey.; II MD. Assistant Professor, Department of Children and Adolescents, İzmir Katip Celebi Üniversitesi, İzmir, Turkey.; III MD. Professor, Department of Children and Adolescents, Dokuz Eylül Üniversitesi, İzmir, Turkey.

**Keywords:** Anxiety disorders, Child, Family, Quality of life

## Abstract

**BACKGROUND:**

Studies have shown that children with anxiety disorders (ADs) present impaired family functioning and quality of life. We aimed to evaluate family functioning and quality of life among children with AD and healthy controls.

**DESIGN AND SETTING:**

Cross-sectional study (survey) at two centers in Turkey.

**METHODS:**

The study group comprised 42 children diagnosed with AD and 55 controls. The Screen for Child Anxiety-Related Emotional Disorders (SCARED) questionnaire was filled out by their parents to measure the severity of anxiety symptoms. Family functioning among the children was assessed using the Family Assessment Device (FAD) and Parental Attitude Research Instrument (PARI). The children’s quality of life was assessed through the Pediatric Quality of Life Inventory (PedsQL).

**RESULTS:**

The children’s average age was 10.00 ± 0.21 years in the AD group and 9.98 ± 1.53 years among the controls. There were higher scores on all FAD subscales in the AD group (2.15 ± 0.52; 2.29 ± 0.44; 2.44 ± 0.55; 2.10 ± 0.61; 2.56 ± 0.40; 2.32 ± 0.33; and 2.29 ± 0.47). On PARI subscales, there were significant differences favoring the AD group (p < 0.05), except for democratic attitude. All PedsQL subscales differed significantly between the groups, favoring the AD group. A statistically significant relationship was found between all PedsQL subscales and SCARED scores in the AD group.

**CONCLUSION:**

We found that both family functioning and quality of life among children with AD were negatively affected. However, further studies with larger sample sizes are required to reach stronger conclusions.

## INTRODUCTION

Anxiety disorders (ADs) usually begin in childhood^1^ and they cause serious impairment of academic performance, peer relationships and family functioning.^2^ Early onset of AD tends especially to show a chronic course.^3^ The presence of AD decreases with age and, by late adolescence or early adulthood, secondary psychopathological conditions such as depressive disorders or substance use disorders have frequently developed.^4^

In many studies focusing on adults, it has been shown that AD has a negative impact on quality of life.^5^^,^^6^^,^^7^ Kang et al. evaluated the quality of life among individuals with panic disorder. Their findings suggested that evaluation of symptoms along with individual anxiety-related traits should be included in assessments of quality of life among panic patients.^8^ Although many studies have evaluated quality of life among cases of psychiatric disorders during childhood,^9^^,^^10^ only a few studies have evaluated quality of life among cases of childhood AD alone. In a review, it was shown that quality of life assessed through self-reported scales among cases of childhood mental and behavioral disorder is significantly reduced compared with that of healthy controls.^11^ Martinsen et al. evaluated sad and anxious children in terms of quality of life. They found that internalization of symptoms such as depressive and anxious symptoms was associated with lower self-reported quality of life and self-esteem.^12^

In studies examining family functioning and parental attitudes in cases of AD, various features have been included. It has been shown that overprotective attitudes of families may be associated with AD in children.^13^ A reciprocal relationship between parental overprotectiveness and anxiety among offspring has been seen in most models.^14^ Towe Goodman et al. assessed the perceived family impact of preschool anxiety disorders. They found that preschool anxiety had an important, unique impact on family functioning, particularly parental adjustment, thus highlighting the family impairment linked to early anxiety.^15^

It is likely that AD is more common among children whose parents present anxiety.^16^ Parents with anxiety may show more fear and anxiety reactions. This is a risk factor for development of AD in children.^17^ In Turkey, anxiety levels have been investigated among children of both divorced and married parents. It was found that the anxiety scores among children with divorced parents were significantly higher than those among children living with both of their parents.^18^

We aimed to evaluate family functioning and quality of life among children with anxiety disorders (ADs) and among healthy controls.

## METHODS

### Study design, date, setting and ethical issues

This study was a cross-sectional analytical study (survey) with a healthy control group. It was conducted at two centers (Nevşehir State Hospital and Izmir Atatürk Training and Research Hospital), from August 2016 to December 2016. The research protocol was approved by the Research Ethics Committee of Izmir Katip Celebi University of Medical Sciences, on August 11, 2016, under number 223. All participants gave their informed consent to participate in the study. All of the study procedures were in accordance with the Declaration of Helsinki and with local laws and regulations.

### Participants

**Figure 1:**
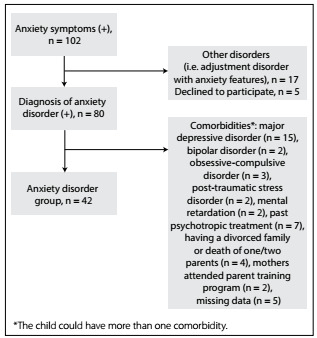
Study flowchart.

The participants for the AD group comprised patients aged 8-12 years with anxiety symptoms who were consecutively admitted to these two centers between August 2016 and October 2016. The inclusion criteria for the AD group were that the subjects needed to: have a diagnosis of AD in accordance with the descriptions in the Diagnostic and Statistical Manual of Mental Disorders, version 5 (DSM-V); be treatment naïve; have learned to read and write in first grade of school and have clinically normal intelligence; be living with both of their parents; be free from chronic medical or neurological conditions requiring treatment (e.g. epilepsy or diabetes etc.); and provide informed consent for study participation. The exclusion criteria for the AD group were situations in which the children had received diagnoses of major depressive disorder, bipolar disorder, psychotic disorders, obsessive-compulsive disorder, post-traumatic stress disorder (PTSD) or mental retardation; the children were using psychotropic medication; the children had a divorce in the family or one/both parents had died; the mothers had undergone a parent training program; or the children had medical and neurological disorders. The children and their mothers were evaluated by the same child psychiatrist. The study flow chart for the AD group is shown in Figure 1.

The healthy control group was formed after the participants in the AD group had been chosen. It was composed of children aged between 8 and 12 years who were chosen from the pediatric clinics of the two centers between October 2016 and December 2016. Pediatricians identified children aged 8-12 years and asked the parents whether they would be interested in participating in the study. The inclusion criteria for participants in the healthy control group were that they needed to have come to either of the pediatric clinics with non-psychiatric symptoms between October 2016 and December 2016 and to be living with both of their parents. The exclusion criteria for the healthy control group were situations in which the subjects presented psychiatric disorders and were using psychotropic medication; the subjects had a divorce in the family or one/both parents had died; the subjects’ mothers had undergone a parent training program; the subjects had chronic medical and neurological disorders; or the subjects had been admitted to a psychiatric clinic.

### Data collection

#### Schedule for Affective Disorders and Schizophrenia for School Age Children Present and Life-time KIDDIE-SADS-PL 

The comorbidities of the children in the AD group were examined by means of the Schedule for Affective Disorders and Schizophrenia for School-Age Children Present and Life-time (K-SADS-PL). The K-SADS-PL instrument is applied in the form of a semi-structured diagnostic interview that is designed to assess current and past episodes of psychopathological conditions in children and adolescents, in accordance with the DSM-III-R and DSM-IV criteria. Child and parent ratings are combined in a compound summary.^19^

The questionnaire consists of three sections: the questions in the first section seek information on sociodemographic characteristics, the second section asks about current and past episodes of psychiatric symptoms and the third section evaluates the general functions of the children during the evaluation. Mood disorders, psychotic disorders, anxiety disorders, elimination disorders, disruptive behavior disorders, alcohol and drug use disorders, eating disorders and tic disorders are evaluated during the interview. It is administered by psychologists or child psychiatrists to both the children and their parents and produces a score that takes into account all the data collected from the various sources available (family, children, teachers, pediatricians, etc.).^19^

The Turkish translation of K-SADS-PL and a validity and reliability study on this translation were performed by Gökler et al.^20^

#### The Screen for Anxiety-Related Emotional Disorders (SCARED):

The SCARED scale was used to indicate the degree of veracity of descriptive phrases regarding how children may have felt over the course of the previous three months.

This scale is a 41-item standardized screen for anxiety that has been validated for use among children aged 8 to 16 years and also features a parent report form.^21^ The child version, or self-report form, is answered by the child with regard to his/her own anxiety. The parent-reported SCARED form is completed by parents with regard to their child’s anxiety. 

Participants rate the items for each factor on a three-point scale (0 = not true or hardly ever true; 1 = sometimes true; or 2 = true or often true). The total score for the Screen for Child Anxiety-Related Emotional Disorders (SCARED) scale is obtained by summing the responses from the 41 items and can range from 0 to 82. The purpose of the instrument is to screen for signs of anxiety disorders in children. Higher scores indicate greater severity of anxiety. A composite score of 25 or higher suggests the presence of an anxiety disorder.^21^

The validity and reliability of the Turkish version of SCARED was ascertained by Cakmakci.^22^

#### The Pediatric Quality of life Inventory (PedsQL):

The PedsQL scale was used to assess problems within the multidimensional health-related quality of life in the last month.

This is a modular instrument that is designed to measure health-related quality of life (HRQOL) among children and adolescents aged 2 to 18 years.^23^ It is answered by both children and their parents.

The 23-item PedsQL 4.0 Generic Core Scale encompasses the following: 1) physical functioning (8 items); 2) emotional functioning (5 items); 3) social functioning (5 items); and 4) school functioning (5 items). It was developed through focus groups and cognitive interviews. All its items have a five-point response scale (0 = never a problem; 1 = almost never a problem; 2 = sometimes a problem; 3 = often a problem; and 4 = almost always a problem), and they are reverse-scored and linearly transformed into a 0-100 scale, such that higher scores indicate better functioning. Each scale score is computed as the sum of the items divided by the number of items answered on the scale. If more than 50% of the items on a scale are missing, no score is computed.^23^

Cakın-Memik studied the reliability and validity of the Turkish version of PedsQL.^24^

#### Parental Attitude Research Instrument (PARI):

The PARI scale was used to evaluate the parental attitudes towards child rearing.

The questionnaire was developed by Schaefer and Bell in 1958^25^ and consists of five sections. It is completed by parents and aims to rate parents’ child-rearing attitudes. Furthermore, it rates five separate dimensions in the form of “overprotective mothership”, “democratic treatment and granting of equality”, “rejection of housewifery role”, “incompatibility” and “rigid disciplining”. It is a Likert-type scale and each item can be rated from 1 to 4 points. All items except for items 2, 29 and 44 are rated with a directly scored grade. The scores are summed separately for each factor dimension. Thus, five separate scores reflecting five distinct dimensions are calculated for each case instead of a single summed score. High scores for factors other than “democratic treatment and granting of equality” indicate negative parent attitudes. There is no total score but, rather, actor scores are taken into consideration.^25^

The validity and reliability of the scale were ascertained by Küçük.^26^

#### Family Assessment Device (FAD):

The FAD scale was used to determine problems relating to family functioning.

The instrument was developed by Epstein et al., in 1983, and its questions are answered by the parents.^27^ It comprises seven sections, as follows. The first section addresses problem-solving skills; the second, intra-family communication; the third, roles in the family; the fourth, effective responsiveness against emotions such as sadness, anger, fear, joy, love and interest; the fifth, effective involvement of family members; the sixth, behavior control; and the seventh, general functions in the family. This instrument consists of 60 statements about a family, and subjects are required to rate the extent which the description of each statement is concordant with the situation in their own family. All items are rated on a four-point Likert scale on which the response choices range from 1 (strongly agree) to 4 (strongly disagree). Higher scores indicate worse levels of family functioning.

FAD has been widely used in both research and clinical practice. Its uses include: (1) screening to identify families experiencing problems; (2) screening to identify specific domains in which families are experiencing problems; and (3) assessment of changes following treatment.

Bulut et al. translated the Turkish version of this questionnaire and conducted a validity and reliability study on it.^28^

#### Children’s success at school and peer relationships

The children’s level of success at school was assessed based on their final grade point average (80-100 = good; 60-80 = medium; and 0-60 = poor).

Peer relationships were assessed through the children’s declarations, by asking how well they got along with their friends. The children and their families were asked to give responses (yes/no) to the following statements regarding peer relationships: 1. Peers let this child play with them; 2. This child is chosen as a playmate by peers; 3. Peers approach this child; 4. This child is included in peers’ activities; 5. This child is noticed by peers; and 6. This child is much liked by other children. If the response to more than half of the statements was yes, the peer relationships were deemed to be good. If the response to more than half of the statements was no, the peer relationships were deemed to be bad.

### Statistical analysis

The data of this study were evaluated using the Statistical Package for the Social Sciences (SPSS), version 22.0. Continuous variables are presented by means of summary statistics. This (unless otherwise stated) consisted of the number of patients (n), mean and standard deviation (SD). Categorical data are presented using either absolute or relative frequencies. Demographic data were compared using the chi-square test. A continuity correction (Yates’s correction) and Fisher’s exact test were applied when required. The distribution of the data was evaluated using the Kolmogorov-Smirnov method. Since the data demonstrated normal distribution, two-up groups were evaluated by means of the parametric t test and triple groups were evaluated through the analysis of variance (ANOVA) test. Pearson correlation analysis was used to determine the relationships between continuous variables. All tests were two-tailed, and P-values < 0.05 were considered signiﬁcant.

## RESULTS

Within the study period (August 2016 to December 2016), 42 children with AD and 55 healthy control children were enrolled. During this period, 58 healthy children without psychiatric, chronic medical or neurological symptoms came to the two pediatric clinics. Three of them declined to participate in the study. Thus, the healthy control group comprised 55 children.

The average age of the children in the AD group was 10.00 ± 0.21 years, and the average age of the children in the healthy control group was 9.98 ± 1.53 years. No significant difference was found between the average ages of the groups (P = 0.114). Thirty children in the AD group and 44 children in the healthy control group were female. No statistically significant difference was found between the groups in terms of gender (P = 0.346). There were also no differences between the AD and healthy control groups in terms of the mother’s age and educational level (P = 0.066 and P = 0.505, respectively). There were significant differences between the groups in terms of the level of success at school and peer relationships (P < 0.001 and P < 0.001, respectively). The sociodemographic data are presented in Table 1. The AD subtypes in the AD group are presented in Table 2.

**Table 1:** Sociodemographic data of the AD and control groups

	AD group (n = 42)	Healthy control group (n = 55)	P
Age* (mean ± SD)	10.00 ± 0.21	9.98 ± 1.53	0.144
Gender** n (%)			
Male	12 (28.6)	11 (20.0)	0.346
Female	30 (71.4)	44 (80.0)	
Mother’s mean age* (mean ± SD)	39.07 ± 3.99	36.98 ± 6.39	0.066
Maternal education** n (%)			
< 8 years	26 (61.9)	41 (74.5)	0.505
> 8 years	16 (38.1)	14 (25.5)	
School success** n (%)			
Good	29 (69.1)	47 (85.5)	< 0.001
Medium	10 (23.8)	6 (10.9)	
Poor	3 (7.1)	2 (3.6)	
Peer relationship** n (%)			
Good	30 (71.4)	50 (90.9)	< 0.001
Bad	12 (28.6)	5 (9.1)	

AD = anxiety disorder; SD = standard deviation. *evaluated using parametric t test, **evaluated using chi-square test.

**Table 2:** Anxiety disorder subtypes

	n	%
Generalized anxiety disorder	10	23.8
Separation anxiety disorder	9	21.4
Specific phobia	8	19.1
Social anxiety disorder	6	14.3
Panic disorder	3	7.1
Generalized anxiety disorder + specific phobia	2	4.8
Social anxiety disorder + separation anxiety disorder	3	7.1
Panic disorder + specific phobia	1	2.4

The mean scores for the child form of SCARED were 38.57 ± 7.1 (range = 34) in the AD group and 6.18 ± 3.47 (range = 16) in the healthy control group. This difference between the two groups was significant (P < 0.001). The mean scores for the parent form of SCARED were 41.12 ± 6.67 (range = 33) in the AD group and 7.54 ± 3.40 (range = 14) in the healthy control group. This difference between the two groups was also significant (P < 0.001). The comparisons between the AD and healthy control groups using the PedsQL, FAD and PARI subscales are presented in Table 3.

**Table 3:** Comparison of the AD and control group in terms of quality of life, family functioning and parental attitude

Scale	AD group	Healthy control group	P	Cohen’s d	Effect size
PedsQL - child form					
Physical functioning	67.09 ± 9.51	84.50 ± 9.13	< 0.001	-1.87	-0.62
Emotional functioning	52.62 ± 16.76	80.46 ± 7.42	< 0.001	-2.15	-0.73
Social functioning	69.05 ± 17.68	79.71 ± 8.40	< 0.001	-0.77	-0.36
School functioning	70.48 ± 18.90	80.09 ± 10.99	0.018	-0.62	-0.30
Total scale score	64.81 ± 14.24	81.51 ± 4.08	< 0.001	-1.59	-0.62
PedsQL - parent form					
Physical functioning	69.29 ± 20.12	84.27 ± 12.32	< 0.001	-0.90	-0.41
Emotional functioning	52.98 ± 18.15	80.37 ± 7.65	< 0.001	-1.97	-060
Social functioning	66.07 ± 17.62	75.91 ± 7.94	0.001	-0.72	-0.34
School functioning	69.76 ± 17.35	78.00 ± 11.81	0.022	-0.56	-0.27
Total scale score	64.52 ± 14.62	79.61 ± 5.15	< 0.001	-1.38	-0.57
PARI overprotective parenting attitude	41.70 ± 6.52	32.62 ± 5.05	< 0.001	1.56	0.61
PARI democratic attitude	23.14 ± 5.23	23.84 ± 2.89	0.858		
PARI rejection of homemaking attitude	33.71 ± 6.29	30.45 ± 5.05	0.010	0.57	0.27
PARI marital conflict	18.62 ± 4.15	15.11 ± 2.74	< 0.001	1.00	0.45
PARI strict discipline	32.21 ± 6.86	27.82 ± 4.79	0.002	0.74	0.35
FAD problem-solving	2.15 ± 0.52	2.07 ± 0.61	0.143		
FAD communication	2.29 ± 0.44	1.87 ± 0.53	< 0.001	0.86	0.40
FAD roles	2.44 ± 0.55	1.81 ± 0.64	< 0.001	1.07	0.47
FAD affective emotions	2.10 ± 0.61	1.67 ± 0.49	< 0.001	0.78	0.36
FAD affective attachment	2.56 ± 0.40	1.77 ± 0.51	< 0.001	1.72	0.65
FAD behavior control	2.32 ± 0.33	1.74 ± 0.64	< 0.001	1.14	0.49
FAD general functionality	2.29 ± 0.47	1.81 ± 0.45	< 0.001	1.04	0.46

AD = anxiety disorder; PedsQL = Pediatric Quality of Life Inventory; PARI = Parental Attitude Research Instrument; FAD = Family Assessment Device.

In comparing the PedsQL scores with the child and parent SCARED scores, significant negative correlations were found between both the child and parent SCARED scores and all the subscales of PedsQL (PedsQL school functioning subscale P = 0.028; and other subscales P = < 0.001) (Table 4). When the control group was compared in the same way, there was no significant correlation between the child SCARED scores and the PedsQL subscales (P > 0.05).

**Table 4:** Examination of the relationship of child and parent SCARED scores with children’s quality of life scores in the AD group (Spearman correlation analysis)

		PedsQL PF	PedsQL EF	PedsQL SF	PedsQL ScF	PedsQL Total score
SCARED - child form	r	-0.541	-0.424	-0.447	-0.380	-0.561
	P	< 0.001	< 0.001	< 0.001	0.028	< 0.001
SCARED - parent form	r	-0.474	-0.528	-0.453	-0.276	-0.550
	P	< 0.001	< 0.001	< 0.001	0.076	< 0.001

SCARED = Screen for Anxiety-Related Emotional Disorders; AD = anxiety disorder; PedsQL = Pediatric Quality of Life Inventory; PF = Physical functioning; EF = Emotional functioning; SF = Social functioning; ScF = School functioning.

## DISCUSSION

This study aimed to compare family functioning, parental attitudes and quality of life between AD and healthy control groups. We found that the children with a diagnosis of AD who had not yet been started on medication had more difficulty in family functioning and in relation to parental attitudes than did the children without any psychiatric diagnosis or chronic disease. Furthermore, we found that impairment of quality of life was more prominent among the children with a diagnosis of AD than among the controls. The findings from our study are similar to those of previous studies examining relationships between AD and family functioning and between AD and parental attitudes.^29^^,^^30^^,^^31^

Studies evaluating links between AD and family relationships have drawn attention both to familial risk factors for development of AD and to the attitudes of the family members of children with AD. In a meta-analysis within a study that evaluated parental factors associated with anxiety among young people, it was shown that the parental factors that gave rise to increased risk of anxiety included less warmth, more inter-parental conflict, over-involvement and aversiveness.^31^ Furthermore, in the Duke Preschool Anxiety Study on preschoolers (ages 2-5 years), 917 parents were evaluated regarding the perceived impact of families on preschool children with AD. These parents were interviewed using the Preschool Age Psychiatric Assessment,^32^ an interviewer-based diagnostic assessment for two to five-year-olds. It was found that preschool anxiety had an important, unprecedented effect on family functioning, particularly parental adjustment, thus highlighting the family impairment that was linked with early anxiety.^15^ In the same study, it was found that generalized AD and separation AD were similar to the impaired family functioning in ADHD.^15^

Another finding in our study was that children with AD showed greater deterioration than healthy controls in all areas of the PedsQL, as assessed through self-reports both from the children and from their families. While there are many studies evaluating quality of life among adults with AD, the limitation of similar studies regarding childhood anxiety disorder is notable. In a study evaluating quality of life among individuals with panic disorder, which is a subtype of anxiety disorder, quality of life was assessed using the short form-36 scale,^33^ which is similar to the PedsQL scale. Consequently, anxiety sensitivity and anxiety traits were found to be independent determinants of quality of life. Therefore, it was suggested that evaluation of quality of life in cases of panic disorder should include evaluation of the symptoms of the disease.^8^ Weitkamp et al. evaluated 120 patients as part of an effectiveness trial for child and adolescent psychotherapy in Germany. They aimed to demonstrate a relationship between childhood mental disorders (45.1% with an anxiety disorder, 31.0% with an affective disorder, 25.7% with a PTSD, 15.9% with a disruptive disorder, and 33.6% with other disorders) and the quality of life, as assessed using the German Kidscreen,^34^ which is similar to PedsQL. They reported that impairment of the quality of life was strongly associated with internalizing rather than externalizing pathological conditions, according to both self-reports and parental reports, and they also found a relationship between mental disorders and impairment of the quality of life.^35^ In another study assessing 310 children (ages 6-18 years) at an outpatient child psychiatric clinic in Rotterdam, Netherlands, who had been referred because of psychiatric problems, the aim was to determine the relationship between the most prevalent child psychiatric diagnoses and quality of life measured through PedsQL. It was found that the overall quality of life of children diagnosed with psychiatric disorders, including AD, was more impaired than that of healthy controls.^10^ The findings from our study and data in the literature suggest that the quality of life of children with AD is impaired significantly.

In our study, there was a significant negative correlation between anxiety symptom severity and all the quality of life sub-scores. This negative correlation showed that as AD symptom severity increased, quality of life became more impaired. In a study conducted by Ramsawh and Chavira in 2016, 73 children (aged 8-12 years) from district pediatric primary care practices were evaluated as part of a larger study focusing on mental health service utilization. This pediatric primary care sample was used to examine the relationships between child anxiety and quality of life measured using PedsQL. It was found that having more than one diagnosis of comorbid AD and having greater severity of anxiety symptoms were associated with reduced quality of life.^36^ It has also been shown in studies that quality of life problems originating from AD can be improved through appropriate treatment. For example, Memik et al. (2014) studied the effects of sertraline on the quality of life of children and adolescents with AD and found that sertraline treatment improved the quality of life.^37^

There are some limitations to our study. First, the mothers’ psychiatric status was not assessed. Second, our sample size may have limited the generalizability of our findings. Third, the children and their mothers were evaluated by the same child psychiatrist, which therefore gave rise to lack of blinding. Fourth, only using information from the mothers in our study may have affected the objectivity of our study. Information from the children’s teachers might have provided greater objectivity for the results from the study. The children’s mental capacity could have been assessed through objective tests. The data analyzed here were obtained before treatments were implemented for the children in this study; the changes achieved through the treatment could be examined in the future.

## CONCLUSION

We found that both family functioning and the quality of life of children with AD were negatively affected and that, as the severity of anxiety symptoms increased, the quality of life of the children with AD diminished. Consequently, we consider that it is important to address family functioning and quality of life when planning AD treatments for children.
